# Green-synthesized copper nanoparticles as a potential antifungal against plant pathogens

**DOI:** 10.1039/c9ra03110c

**Published:** 2019-06-14

**Authors:** Nicolaza Pariona, Arturo I. Mtz-Enriquez, D. Sánchez-Rangel, Gloria Carrión, F. Paraguay-Delgado, Greta Rosas-Saito

**Affiliations:** Red de Estudios Moleculares Avanzados, Instituto de Ecología A. C. Carretera Antigua a Coatepec 351, El Haya 91070 Xalapa Veracruz Mexico conipariona@gmail.com nicolaza.pariona@inecol.mx; Centro de Investigación y de Estudios Avanzados del IPN Unidad Saltillo Av. Industria Metalúrgica 1062, Parque Industrial Ramos Arizpe 25900 Coahuila Mexico arturo.martinez@cinvestav.edu.mx; Cátedra CONACYT en el Instituto de Ecología A. C. 91070 Xalapa Mexico; Red de Biodiversidad y Sistemática, Instituto de Ecología A. C. Carretera Antigua a Coatepec 351, El Haya 91070 Xalapa Veracruz Mexico; Centro de Investigación en Materiales Avanzados SC (CIMAV), Laboratorio Nacional de Nanotecnología Miguel de Cervantes No. 120 C. P. 31136 Chihuahua Mexico

## Abstract

The fabrication of fungicides in cost-effective and eco-friendly ways is particularly important for agriculture. Plant pathogenic fungi produce many economic and ecological problems worldwide, which must be controlled with potent fungicides. Here we propose the green synthesis of fungicides, which consist of copper nanoparticles (Cu-NPs) prepared in aqueous media. Through *in vitro* experiments, the antifungal efficacy against *Fusarium solani*, *Neofusicoccum* sp., and *Fusarium oxysporum* was investigated. Although the antifungal activity differs for each fungal species, it was found that the Cu-NPs induce strong morphological changes in the mycelium. Additionally, the damage of the cell membranes of the pathogens was revealed by microscopic observations. For the three evaluated fungi, fluorescence microscopy demonstrated the intracellular generation of reactive oxygen species in the mycelium. This work proves that the green-synthesized Cu-NPs are potential fungicides against *F. solani*, *Neofusicoccum* sp., and *F. oxysporum*.

## Abstract 2

The fabrication of fungicides in cost-effective and eco-friendly ways is particularly important for agriculture.

## Graphical abstract

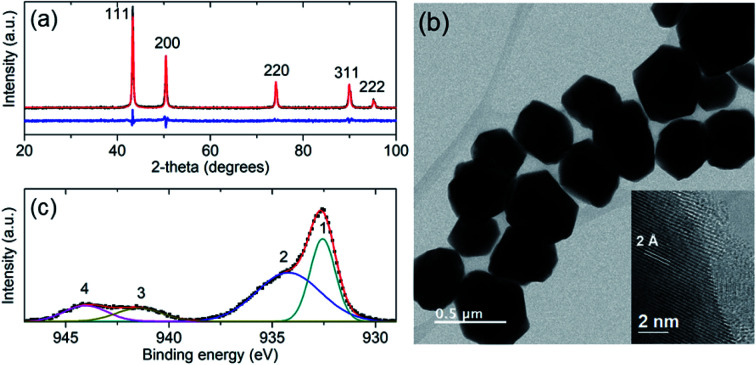

## 1. Introduction

Pathogenic fungi cause a range of diseases on a variety of agronomic, horticultural, ornamental, and forest plant species.^1,2^ Among the methods for the control of plant pathogens, the use of site-specific fungicides is the most prevalent.^3^ However, the target fungi can develop different defenses to pesticides; it means that plant pathogenic fungi can adapt to fungicides by mutations, leading to the loss of fungicide efficacy.^3,4^ For many decades, copper ions have been used as pesticides, fungicides, and fertilizers.^5^ For example, some copper salts have been used for reducing mildew in grapes.^5^ However, the use of copper ions presents some disadvantages, such as instability in field conditions, high solubility in water, and formulations with high concentrations of copper salts. Therefore, it is necessary to develop long-term efficient, eco-friendly, and cost-effective fungicides.

In the last years, nanotechnology has proposed different tools for solving agricultural problems. It has been reported that nanotechnology may improve the production of the crop with the development of nano-fertilizers and nano-treatments for plant diseases.^6–8^ Different nanoparticles (NPs) have been studied for the treatment of plant diseases.^9^ Particularly, it has been claimed that copper (Cu) NPs can be a potent fungicide. For agricultural applications, the Cu-NPs should be synthesized in a cost-effective way. There are many synthesis methods such as hydrothermal, microwave, photochemical, electrochemical, microemulsion, and chemical reduction.^10,11^ Nonetheless, most of them use harmful chemicals, use critical reaction conditions, organic solvents, and expensive equipment. In this sense, for the synthesis of stable Cu-NPs a green synthesis method that uses environmentally friendly and cost-effective chemicals should be proposed. Here we propose a synthesis method that use ascorbic acid as a reducing agent of Cu^2+^ ions. In addition, ascorbic acid was used as stabilizing agent, these chemicals are massively used in the food industry. To the best of our knowledge, both chemicals have not been used jointly for the preparation of water dispersible Cu-NPs. In contrast, some reports have used citrate and poly(vinyl alcohol) as stabilizing agent^12^ and only ascorbic acid as reducing agent.^13^ However, the reported powders exhibited strong agglomeration or micro-sized powders, which cannot be adequate for their application as fungicides.

Some fungi such as *Fusarium solani*, *Fusarium oxysporum*, and *Neofusicoccum* sp. *F. solani* and *F. oxysporum* attack crops and forest species globally, which produce many economic and ecological problems. These fungi invade plant vascular tissues, inhibit water transport through xylem by inducing vessel plugging, and lead to foliage wilt.^14,15^ In addition, *Neofusicoccum* sp. a genus of fungi in the Botryosphaeriaceae family has been associated with canker and dieback symptoms in a broad range of different perennial fruit crops and species of trees.^16,17^ It has been reported that Cu-NPs act as a fungicide against a number of species of plant pathogenic fungi such as *Fusarium* sp., *Phoma destructiva*, *Curvularia lunata*, *Alternaria alternate*, *Fusarium oxysporum*, *Penicillium italicum*, *Penicillium digitatum* and, *Rhizoctonia solani*.^18–21^ To the best of our knowledge, the antifungal activity of Cu-NPs against pathogenic fungi that affect crop and forests species has not been investigated fully. Here we present the antifungal properties of Cu-NPs against three species of plant pathogenic fungi. Firstly, we describe the characterization of the green synthesized Cu-NPs which are stable in aqueous media. This research shows that the Cu-NPs are stable in water and have potential antifungal activity against *F. solani*, *F. oxysporum*, and *Neofosicoccum*.

## 2. Materials and methods

### 2.1. Synthesis and characterization of the copper nanoparticles

All the chemicals were of analytical grade and were used without further purification. The Cu-NPs were synthesized as follows: 2.5 g of sodium citrate tribasic dihydrate was dissolved in 100 mL of distilled water and then 5 g of copper(ii) sulfate pentahydrate was added. Next, 50 mL of a solution of ascorbic acid (0.2 M) and 30 mL of a solution of sodium hydroxide (1 M) were added under stirring. Afterward, the mixture was heated at 95 °C for 90 min at the open atmosphere. After this time, a precipitate red in color is formed, which indicates the formation of Cu-NPs. Using centrifugation, the precipitate was washed three times with distilled water and one time with ethanol. The obtained powder was dried at room temperature for 48 h.

The crystal structure of the Cu-NPs was characterized by X-ray diffraction (XRD) using a Bruker X-ray diffractometer, operating in the Bragg–Brentano geometry and equipped with a Cu-anode X-ray source (Kα, *λ* = 0.15418 nm). The patterns were collected with a scan rate of 0.04° s^−1^ in the 20–90° 2*θ* range. The XRD patterns were indexed using the powder diffraction files (PDF) database, and Rietveld refinement was done using the MAUD program v. 2.33.^22^ Transmission electron microscopy (TEM) studies were done in a JEOL JEM-2000EX microscope with an accelerating voltage of 200 kV. Samples for TEM measurements were suspended in ethanol and ultrasonically dispersed. Then drops of the suspensions were placed on a nickel grid coated with carbon and the solvent was evaporated prior to TEM observations. The X-ray photoelectron spectroscopy (XPS) analysis was performed with a model K-Alpha equipment from Thermo Scientific Instruments, which employed a monochromatic Al Kα radiation (*E* = 1486.68 eV) with a resolution of 0.1 eV.

### 2.2. Assessment of antifungal activity of Cu-NPs on mycelial radial growth

The antifungal activity of the Cu-NPs was evaluated against *Fusarium solani* (strain INECOL_BM-04), *Neofusicoccum* sp. (strain INECOL_BM-03), and *Fusarium oxysporum* (strain INECOL_CBF-185). Those species were kindly provided by the Laboratories of Molecular Biology and Biologic Control of the Institute of Ecology A. C., Xalapa, Veracruz, Mexico. To determine the inhibition of mycelial growth of the fungi, the fungal samples were incubated in potato dextrose agar (PDA) mixed with different amounts of Cu-NPs. The final concentrations of Cu-NPs in the growth media were: 0, 0.1, 0.25, 0.5, 0.75 and 1.0 mg mL^−1^. Finally, suspensions of 1 × 10^6^ CFU mL^−1^ cells were inoculated at the center of each fresh PDA solid media, followed by the incubation at 29 °C for 6 days. PDA plates without Cu-NPs were used as controls, using the same grown conditions for each pathogenic fungus. Each treatment was carried out in triplicates. The photographic record and the radial colony growth were measured after 6 days and the percentage of fungal inhibition was calculated based on the percent inhibition of radial growth (IRG) as a follow:
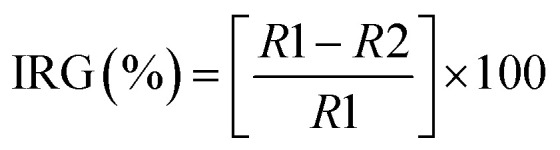
where *R*1 is the radial growth of the control and *R*2 is the radial growth for each treatment. The IRG data were analyzed using the mean standard deviation.

### 2.3. Analysis of fungal morphology through field emission scanning electron microscopy

The growth morphology was studied in six-day-old fungal cultures for the three species (controls and the Cu-NPs treatments). For these studies, the mycelial discs were cut from the peripheral in a 10 × 10 mm^2^ area and fixed with 2.5% glutaraldehyde at 4 °C for 24 h. Subsequently, the samples were washed with Sorensen phosphate buffer three times for 1 min. Then, the samples were dehydrated in a graded series of ethanol, started with gradual dehydration in ethanol from 30, 40, 50, 60, 70, 80 to 90% for 40 min in each aqueous solution. The last step was performed in 100% ethanol for 30 min thrice. The dehydrated samples were dried in a Quorum K850 critical point dryer and placed in aluminum specimens on double adhesive carbon conductive tape and finally gold-coated for one minute in a Quorum Q150R S coater.^23^ Finally, the samples were observed in a scanning electron microscope (SEM) FEI Quanta 250 FEG.

### 2.4. Analysis of viability through confocal microscopy

To investigate the effect of Cu-NPs on cell viability, propidium iodide (PI) was used. It has been reported that PI forms a bright red fluorescent complex with DNA and RNA in the nuclei of dying or dead cells but not in healthy cells, it is because PI overcome damaged cell membranes.^24^ The viability was studied in six days old mycelia for the fungi treated with 0.5 mg mL^−1^ of Cu-NPs. Firstly, each mycelium was re-suspended in 1 mL of Tris-KCl solution and later treated in 1 mL of PI (final concentration of 5 μg mL^−1^) for 10 min under dark conditions. Stained cells were washed thrice with a Tris-KCl solution. On the other hand, to show the accumulation of reactive oxygen species (ROS) in the mycelia, the 2′,7′-dichlorofluorescein diacetate (DCFH-DA) assay was conducted. DCFH-DA reacts with ROS to produce a bright green fluorescence. Fluorescence images were observed using confocal laser scanning microscopy (CLSM) using a Leica TCS SP8 microscope, where the confocal setup is based on a DMI6000 inverted microscope. The images were taken using an excitation/emission wavelength of 536/617 and 488/530 nm for IP and DCFH-DA tests, respectively.

### 2.5. Statistical analysis

All fungal growth assays were carried out in triplicate and the data are presented as the mean standard deviation. One-way analysis of variance (ANOVA) was performed to compare the treated group and the controls (without Cu-NPs), followed by Tukey's *post hoc* tests using Origin 9.0 software. Statistical differences were considered significant at *p* < 0.05.

## 3. Result and discussion

### 3.1. Characterization of the Cu-NPs

For agriculture and forestry applications it is necessary to fabricate vast amounts of Cu-NPs, which should be stable in the open atmosphere. Additionally, for the application in plants and trees, the Cu-NPs should be water dispersible. It is a challenge to fabricate Cu-NPs with those characteristics by a green method. Different ways have been employed for the synthesis of Cu-NPs, among them, the chemical reduction techniques are the most prominent. These techniques consist in the reaction of copper ions with reducing agents, such as sodium borohydride, hydrazine, ascorbate, polyol, isopropyl alcohol, glucose, among others.^11,25^ For the fabrication of Cu-NPs at large scales in a green way, the reducing agent should be non-toxic, cheap, stable and water soluble. For these reasons, the ascorbic acid was selected as an effective reducing agent. Furthermore, for the stabilization of Cu-NPs in water dispersions, sodium citrate was used as a dispersant and protecting agent.

**Fig. 1:**
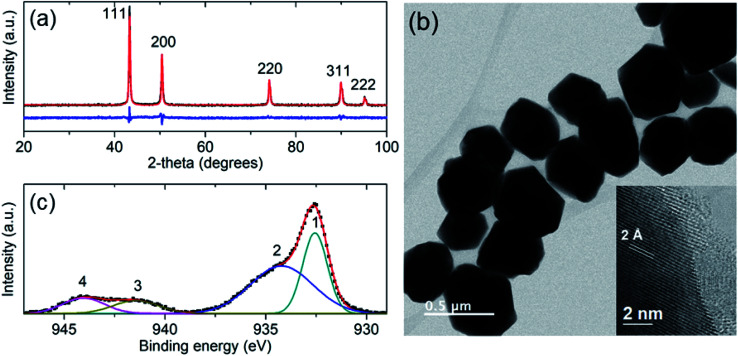
(a) X-ray diffraction pattern of Cu-NPs. (b) TEM micrograph of the Cu-NPs, the inset shows a high resolution TEM image of the border of a Cu-NP. (c) The fitted XPS spectrum of the Cu-NPs.

The structural characterization of the Cu-NPs was studied by complementary techniques such as XRD and high resolution TEM. The XRD pattern of the Cu-NPs in Fig. 1a shows that all the detected diffractions can be indexed to the crystal structure of zero valence copper (space group *Fm*3̄*m*, PDF number 4-836). The structural refinement yielded a cell parameter *a* = 3.612 Å, which is near to the reported standard of *a* = 3.615 Å. Additionally, the morphology of the Cu-NPs was characterized by TEM. Fig. 1b shows a typical TEM image with faceted particles of 200 to 500 nm in size. The inset of Fig. 1b shows the high resolution TEM image of the border of one Cu-NP, where an interplanar distance of 2 Å is observed, which correspond to the {111} planes of the cubic crystal structure of copper. In addition, the XPS spectrum of the Cu-NPs is shown in Fig. 1c. The Cu-2p_3/2_ spectrum shows four bands: (1) at 932.6 eV with a FWHM of 1.5 eV, which has been assigned to either Cu^0^ or Cu^+^;^26^ (2) the centered at 934.2 eV (FWHM = 3.6 eV) assigned to Cu^2+^; and the shakeup satellites (bands 3 and 4 in Fig. 1c) which are characteristics of Cu^2+^ materials.^26^ With the XPS analysis it was found that the surface of the prepared Cu-NPs is formed by different species such as Cu^0^, Cu^+^ and Cu^2+^. These structural and morphological analyses of the Cu-NPs prove that the preparation methodology is fast, cheap, high yield, environmentally safe and can be scalable. Those characteristics are important for the use of Cu-NPs as a fungicide in agriculture and forestry.

### 3.2. Antifungal activity

**Fig. 2:**
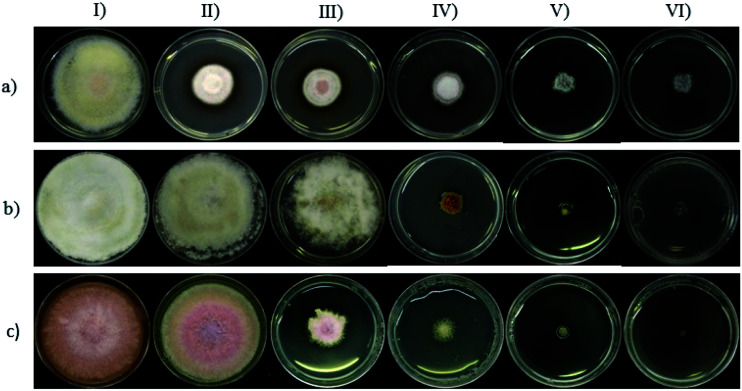
Antifungal activity of Cu-NPs against: (a) *Fusarium solani*, (b) *Neofusicoccum* sp. and (c) *Fusarium oxysporum*. The columns indicate different concentrations of Cu-NPs: (I) 0 (controls), (II) 0.1, (III) 0.25, (IV) 0.5, (V) 0.75 and (VI) 1.0 mg mL^−1^ of Cu-NPs.

**Fig. 3:**
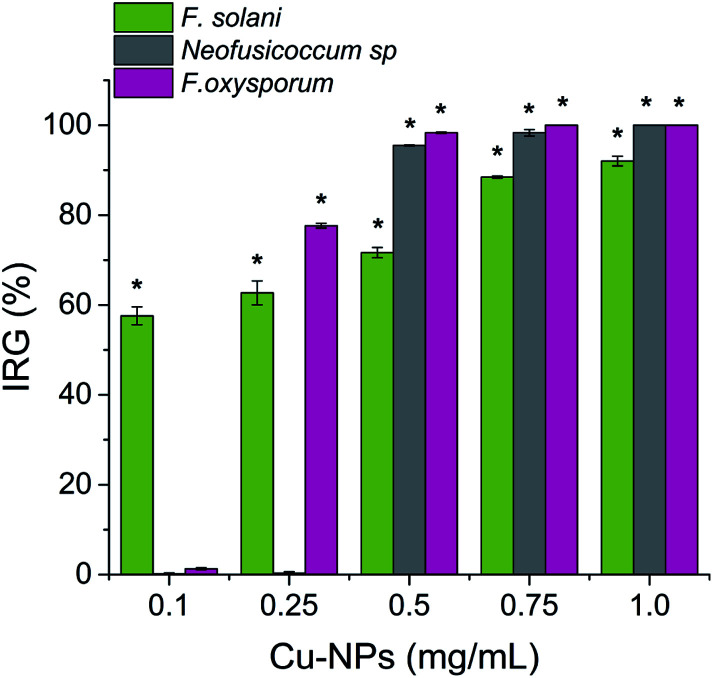
Inhibition of radial growth (IRG) (%) of Cu-NPs against *Fusarium solani*, *Neofusicoccum* sp., and *Fusarium oxysporum*. The quantitative data are expressed as the mean ± SD (*n* = 3). (*) indicates significant differences (*P* < 0.05) in comparison to their controls (treatments without Cu-NPs).

The antifungal activity of the synthesized Cu-NPs was evaluated by measuring the mycelial radial growth for all the treatments. For an incubation period of 6 days, Fig. 2 shows the inhibition effect of Cu-NPs at different concentrations against *Fusarium solani*, *Neofusicoccum* sp., and *Fusarium oxysporum*. It can be observed that the antifungal activity of the Cu-NPs depends on the pathogenic fungi. Similar results have been reported for the antifungal activity of copper nanoparticles against different fungi.^19,27^ Higher concentrations of Cu-NPs in the growth media promote greater IRG (smaller colony areas), see Fig. 2. Similarly, the Cu-NPs change the morphology, color, form, texture, and density of the fungal mycelium. Fig. 3 shows a bar graph of the IRG of Cu-NPs against the evaluated fungi. Fig. 3 indicates that *F. solani* is the most affected fungus at low Cu-NPs concentrations. For example, at 0.1 mg mL^−1^, the IRG was 55%, while the other two fungi species were not affected at this concentration. As shown in Fig. 3, the sensitivities of the fungal species to Cu-NPs were different, this also has been reported for Cu-NPs of 8 nm in size against *F. solani* and other three fungal species.^28^

Fig. 2 and 3 shown that *Neofusicoccum* sp. exhibited high tolerance to Cu-NPs at low concentrations. In contrast, concentrations as high as 0.5 mg mL^−1^ inhibited the growth of the fungus with IRG greater than 95%. To the best of our knowledge, this is the first report about the growth inhibition of *Neofusicoccum* sp. by Cu-NPs. These findings are important because this fungus has been associated with canker and dieback symptoms in a broad range of perennial fruit crops.^17^ On the other hand, concentrations of Cu-NPs as high as 0.5 mg mL^−1^ encourage an IRG greater than 97% for *F. oxysporum*. High antifungal activity against to *F. oxysporum* has also been reported for Cu-NPs of 3–10 nm in size with a cetyl trimethylammonium bromide coating.^21^ In contrast, it has been reported that microparticles of copper-containing metal–organic frameworks at concentrations as high as 0.5 mg mL^−1^ caused 50% of inhibition of *F. oxysporum*.^19^ Summarizing, concentrations of the green-synthesized Cu-NPs higher than 0.75 mg mL^−1^ inhibit almost completely the growth of the three evaluated fungi with IRG near the 100%. As a sake of comparison, for the growth inhibition of *F. solani*, 1.2 mg mL^−1^ of chitosan NPs (size 200–433 nm) were needed.^29^ It can be concluded that the green-synthesized Cu-NPs present high *in vitro* antifungal efficacy against *F. solani*, *Neofusicoccum* sp., and *F. oxysporum*. The great antifungal activity of the Cu-NPs could be attributed to both adequate particle size and the coating used in the synthesis process.

### 3.3. Effect on the fungal morphology

**Fig. 4:**
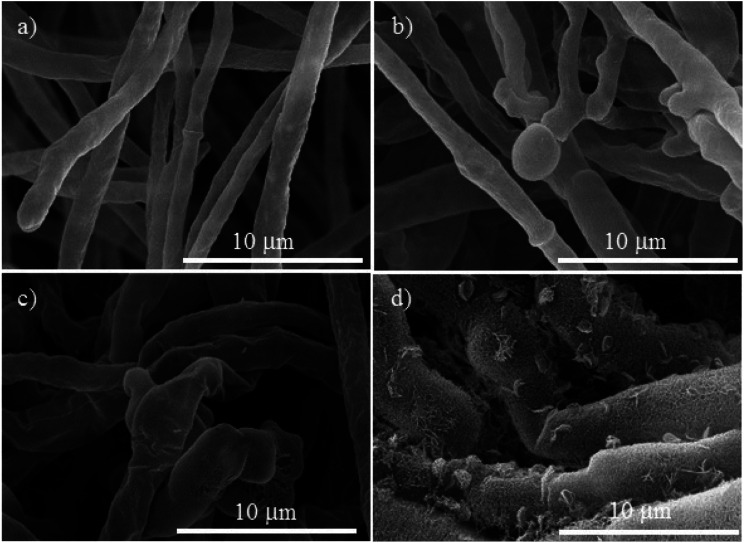
SEM micrographs of *Fusarium solani*, (a) control. The treatments with different concentrations of Cu-NPs: (b) 0.1 mg mL^−1^, (c) 0.25 mg mL^−1^, and (d) 0.5 mg mL^−1^.

**Fig. 5:**
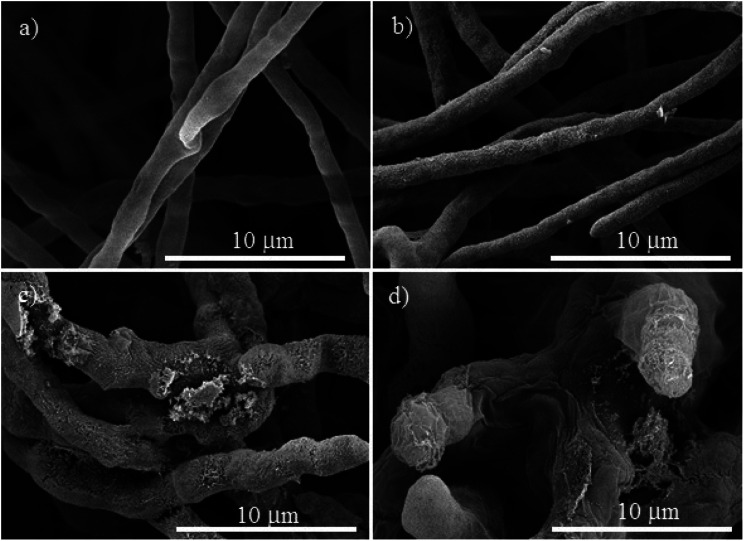
SEM micrographs of *Neofusicoccum* sp., (a) control. The treatments with different concentrations of Cu-NPs: (b) 0.1 mg mL^−1^, (c) 0.25 mg mL^−1^, and (d) 0.5 mg mL^−1^.

**Fig. 6:**
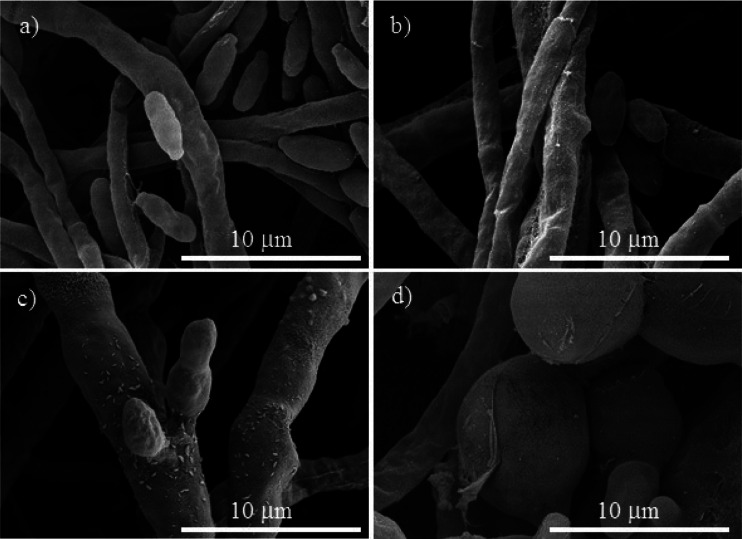
SEM micrographs of *Fusarium oxysporum*, (a) control. The treatments with different concentrations of Cu-NPs: (b) 0.1 mg mL^−1^, (c) 0.25 mg mL^−1^, and (d) 0.5 mg mL^−1^.

The morphological changes on the mycelium for the controls and the Cu-NPs treatments are shown in Fig. 4–6. For *F. solani*, the control shows a normal structural characteristic, which includes a smooth exterior surface on the cylindrically shaped mycelium, see Fig. 4a. In contrast, the Cu-NPs treatments promote deformation of the mycelium, see Fig. 4b–d. A concentration of 0.1 mg mL^−1^ of Cu-NPs formed unusual bulges on the surface of the mycelium, see Fig. 4b. Higher concentrations caused stronger morphological alterations of the mycelium; at 0.25 mg mL^−1^, the mycelium lost their smoothness and is strongly deformed; additionally, many fractures are generated, see Fig. 4c. For 0.5 mg mL^−1^, a rough and peeled mycelium surface is shown in Fig. 4d, it indicates considerable damage of the cell wall, which promotes the outflow of intracellular components and shrinkage of hyphae.

For *Neofusicoccum* sp., the control in Fig. 5a shows a normal mycelium with a cylindrical shape and a smooth exterior. For a concentration of 0.1 mg mL^−1^ of Cu-NPs, an irregular exterior of the mycelium is observed in Fig. 5b. The damage of the mycelium was enhanced for higher concentrations. For concentrations of 0.25–0.5 mg mL^−1^, severe damages are observed, with disruptions of the membrane surface and ruptured mycelium walls, which led to the outflow of the cellular components, see Fig. 5c and d. The effect of Cu-NPs on the mycelium morphology of *F. oxysporum* is shown in Fig. 6. For the control, a smooth surface of the mycelium and a clear conidiation is observed in Fig. 6a. Meanwhile, for 0.1 mg mL^−1^ of Cu-NPs, hyphae filament shaped deformations with irregular shrinkages of mycelium are seen in Fig. 6b. On the other hand, a concentration of 0.25 mg mL^−1^ induced considerable morphological changes on the mycelium, which arise abnormal rough shapes with granules on the surfaces, see Fig. 6c. The damage was intensified with 0.5 mg mL^−1^ of Cu-NPs, where some large vesicles of non-germinated conidia are seen in Fig. 6d, which indicate the inhibition of the fungus growth.

For all the Cu-NPs treatments, different levels of damage were observed in hyphae and cell walls. It is well known that any induced stress affects mainly the fungal cell walls. Fungal cell walls have a complex and dynamic structure and are mainly composed of polysaccharides. Cell walls play an essential role in defining cell shape; also, cell walls shield the cells from environmental stress, including changes in osmolality, temperature, and pH.^30^ The chemical composition of cell walls is different for each fungus;^31,32^ however, their cell walls are primarily composed of chitin, glucans, mannans, and glycoproteins.^33^ Chitin is a structurally important component of the fungal cell walls; when chitin synthesis is disrupted, the wall becomes disordered and the fungal cells become malformed and osmotically unstable.^33^ The SEM images above show the deformed mycelium caused by the treatments with Cu-NPs, which can be attributed to the disruption of the biosynthesis of chitin. It has been suggested that some copper ions are released from the Cu-NPs to the growth medium, which are able to diffuse into the fungal cells and bind tightly on the surface of their cell walls.^34,35^

Given that the composition of fungal cell walls varies between the studied species, it can be expected that the oxidative stress induced by the Cu-NPs acts in a different way for each fungus species. This explains dissimilar inhibition effects in each fungal species at low concentrations of the Cu-NPs. For example, at low concentration of Cu-NPs (0.1 mg mL^−1^), the induced oxidative stress is counteracted by the fungi defenses that recover the redox balance without growth inhibition.^35^ However, the SEM images demonstrated that the mycelia are deformed by the action of low concentrations of Cu-NPs, see Fig. 4b, 5b, and 6b. Thus, the induced antioxidant defense does not avoid completely oxidative stress. The antioxidant defense has been classified as the first level of cellular response to the stress induced by nanomaterials.^36^ Similar effects have been reported for Cu-NPs used against *Aspergillus niger*, *Aspergillus oryzae*, and *Fusarium oxysporum*.^19^

At a concentration of 0.25 mg mL^−1^ of Cu-NPs, *F. solani* and *F. oxysporum* clearly exhibit cell surface damage, such as loss of cell wall integrity and morphological changes that include inflammation of cells (Fig. 4c and 6c). Also, *Neofusicoccum* sp. exhibits loss of cell wall integrity (Fig. 5c), which causes the leakage of cellular components. This demonstrates the direct toxicity mechanism of Cu-NPs, which involves permeation through the cell wall and disturbances in the enzymes involved in the control of free radicals, causing an imbalance to all the metabolic pathways.^37^ It is the second level of the oxidative stress level or pro-inflammatory effects.^36^ Similar results have been observed for silver and copper NPs, which caused detrimental effects on fungal hyphae of some fungi.^38^ On the other hand, at higher concentrations of Cu-NPs (≥0.5 mg mL^−1^), cell death and genotoxic effects were observed for the three plant pathogenic fungi, see Fig. 4d, 5d, and 6d. That is the third level of oxidative stress induced by nanomaterials.^36^ It can be suggested that the Cu-NPs produce ROS, which changes the normal physiological redox-regulated functions. It has been reported that the damage in cell functions produced by ROS generates protein radicals, DNA-strand breaks, disrupted DNA/RNA, free nucleic acids, and modulation of inflammatory responses through signal transduction, leading to cell death and genotoxic effects.^34,36,39^

### 3.4. The cell viability and ROS production

**Fig. 7:**
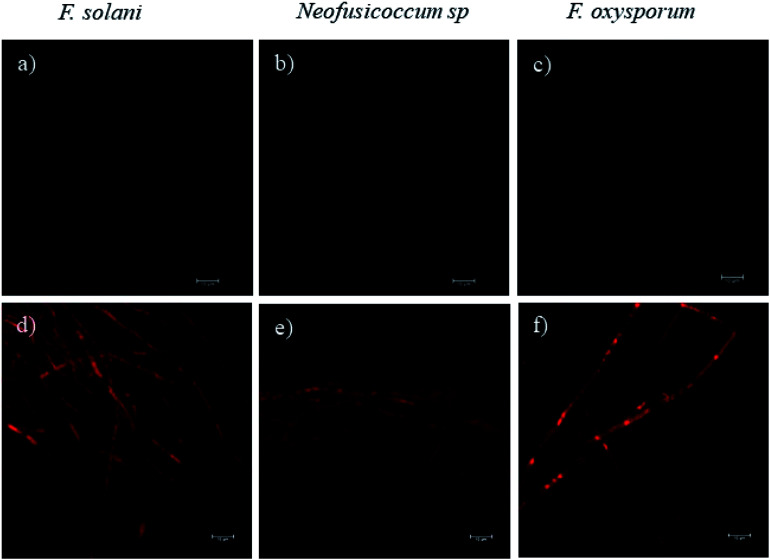
The effect of Cu-NPs on the cell viability by analyzing the PI influx in *F. solani*, *Neofusicoccum* sp., and *F. oxysporum*. (a–c) Controls and (d–f) treatments of Cu-NPs at a concentration of 0.5 mg mL^−1^.

Fluorescence microscopy images obtained by CLSM helped to elucidate the mechanism of growth inhibition induced by the green-synthesized Cu-NPs. The cell viability may be judged by morphological changes, changes in membrane permeability, and/or the physiological state inferred from the exclusion of certain dyes or the uptake and retention of others.^40^Fig. 7 shows the fluorescence microscopy images of the fungi growth in different media, namely, the control and at a concentration of 0.5 mg mL^−1^ of CuNP. The controls for the three fungi do not show any detectable red colored hyphae; see Fig. 7a–c, which mean that all the *F. solani*, *Neofusicoccum* sp. and *F. oxysporum* mycelia were viable. On the other hand, for the samples treated with 0.5 mg mL^−1^ of Cu-NPs, the red colored mycelium is seen in Fig. 7d–f. The uptake of PI by the mycelium suggests that the Cu-NPs damaged the cell membranes, which modify their permeability causing their disintegration and finally the cell death. These findings confirm the SEM observations, which indicated irreversible damage and disintegration of the cell membranes.

**Fig. 8:**
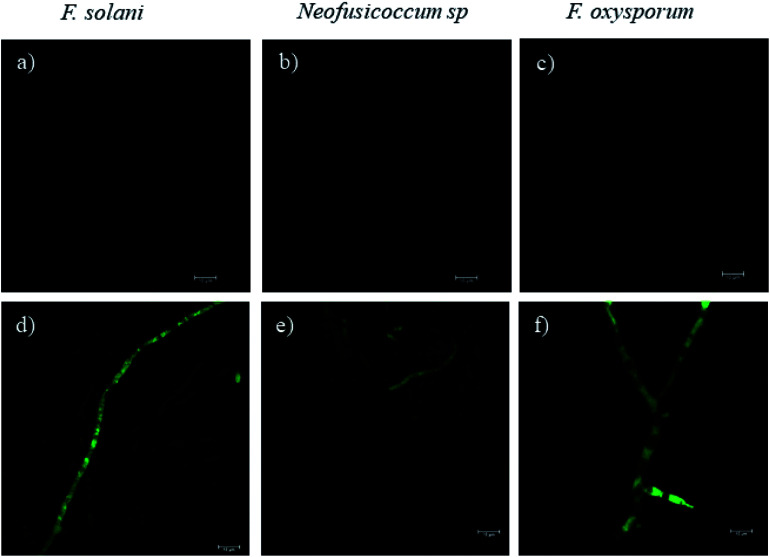
The effect of Cu-NPs on the production of intracellular ROS in *F. solani*, *Neofusicoccum* sp., and *F. oxysporum*. (a–c) Controls and (d–f) treatments of Cu-NPs at a concentration of 0.5 mg mL^−1^.

Metal nanoparticles are described as small particles with a large surface area, which may generate ROS.^36^ There are some reports that proved that the Cu-NPs generate ROS through several mechanisms, such as the Fenton-like and Haber–Weiss reactions; these reactions may take place on the surface of NPs or in dissolved copper ions.^34,41^ The generated ROS induce toxicity, as well as modulation of cellular signaling involved in cell death.^35^ In this study, the DCFH-DA fluorescence was used to study the intracellular ROS production in the fungi mycelium. Fig. 8d–f show the treatments with 0.5 mg mL^−1^ of Cu-NPs, where stronger green colored hyphae are observed for *F. solani*, *Neofusicoccum* sp. and *F. oxysporum*. The micrographs suggest the intracellular ROS generation in the mycelium of the three fungi. In contrast, the fluorescence was not observed in any of the control samples of the fungi, see Fig. 8a–c. In short, it was shown that the green-synthesized Cu-NPs damage the cell membranes and produce intracellular ROS, which inhibit the growth of pathogenic fungi. It has been proved through X-ray absorption spectroscopy that fungus colonies dissolve copper minerals thanks to different ligands, including oxalate, NH_4_^+^, and NO_3_^−^.^42^ Subsequently, the dissolved copper ions produce ROS and oxidize the main components of the fungal cell walls such as glucan and chitin, which causes irreversible damage and disintegration of the cell wall. Once the cell walls are damaged, the Cu-NPs enter to the cells producing ROS that were detected by the DCFH-DA tests.

## 4. Conclusion

Cu-NPs were successfully synthesized by a green method, which consists in the chemical reduction of copper ions with ascorbic acid, using sodium citrate as a protecting agent. These green-synthesized Cu-NPs exhibited high antifungal activity against *F. solani*, *Neofusicoccum* sp., and *F. oxysporum*. Three levels of oxidative stress induced by the Cu-NPs were detected by SEM, which depend on the concentration of the Cu-NPs and fungus species. The mechanism of growth inhibition induced by the green-synthesized Cu-NPs was elucidated by fluorescence microscopy. It was shown that the inhibition involves the damage the cell membranes and the intracellular production of ROS. This work proposes a facile and cheap green-synthesis method for the production of Cu-NPs with high antifungal activity. Thus, this nanomaterial could be useful for controlling pathogenic fungi that affect crop and forest species globally.

## Conflicts of interest

The authors declare no conflict of interest.

## Supplementary Material
